# Association Between Loneliness and the Use of Medical Institutions and Pharmacies in Urban Japan

**DOI:** 10.7759/cureus.76771

**Published:** 2025-01-01

**Authors:** Shinya Sugiura, Akihito Ueda, Michiko Obara

**Affiliations:** 1 Department of Pharmaceutical Sciences, Graduate School of Pharmaceutical Sciences, Teikyo Heisei University, Tokyo, JPN; 2 Department of Internal Medicine, Medical Corporation Toujinkai, Fujitate Hospital, Osaka, JPN; 3 Department of Pharmaceutical Sciences, Faculty of Pharmaceutical Sciences, Teikyo Heisei University, Tokyo, JPN

**Keywords:** loneliness, medical institution use, pharmacy use, public health, ucla loneliness scale

## Abstract

Objective: This study aimed to investigate the association between loneliness and utilization of medical institutions and pharmacies in urban areas of Japan.

Methods: A 52-item survey was distributed to 10,000 residents, aged 15-64 years, in Nakano Ward, Tokyo, Japan. The survey included a three-item version of the UCLA Loneliness Scale, as well as questions on demographic, socioeconomic, and educational background, medical institution and pharmacy use, and community activities.

Results: Of the 3,369 survey respondents, 379 answered “Always feel that way” to at least one of the three items on the loneliness scale. Participants who felt lonely were significantly more likely to be men (p < 0.001), economically disadvantaged (p < 0.001), and perceived their health status as poor (p < 0.001). Loneliness was associated with a significantly lower self-reported frequency of medical institution use (p = 0.003) and pharmacy use (p < 0.001).

Conclusion: Loneliness was associated with male gender, economic disadvantage, poorer perceived health status, and lower self-reported frequency of medical institution and pharmacy use. These findings suggest the existence of barriers preventing lonely people from accessing necessary medical and pharmacy services. Further research is needed to explore these barriers in greater detail.

## Introduction

Loneliness negatively affects health and is a prevalent health issue in rural and urban areas [1]. Individual characteristics play a significant role in explaining the variations in loneliness [2]. Social connection is a critical factor influencing mental and physical health, and its importance has grown in the wake of the coronavirus disease 2019 pandemic [3-5]. A meta-analysis of studies examining the effects of loneliness, social isolation, or living alone on mortality found that both subjective and objective measures of social isolation significantly influence the risk of mortality, comparable to well-established risk factors such as smoking or obesity [6].

However, findings regarding the relationship between loneliness and medical institution use are inconsistent. While some studies report increased medical institution use among those who feel lonely [7,8], others show contrasting findings [9]. Furthermore, to our knowledge, the relationship between loneliness and pharmacy use has not been studied previously. 

This exploratory study aims to investigate the relationship between loneliness and the use of medical institutions and pharmacies in urban areas of Japan.

## Materials and methods

Ethical approval

This study used anonymized data and was conducted in accordance with the Japanese Personal Information Protection Law and the Ethical Guidelines for Epidemiological Research, which do not require individual informed consent for the secondary use of anonymized data. In line with these regulations, ethical committee approval was not required for this study.

Study design and participants

A survey was distributed to 10,000 residents of Nakano Ward, Tokyo, Japan. The inclusion criteria were as follows: residents of Nakano Ward aged 15-64 years who were able to respond to the survey either by mail or online. There were no specific exclusion criteria. All responses meeting these criteria were included in the analysis. Potential participants were selected through random sampling. Respondents had the option to complete the survey either by mail or online. The survey data collection period spanned from December 11, 2020, to January 8, 2021.

In total, 3,369 valid responses were received, resulting in a response rate of 33.7%. No responses were excluded, though some contained unanswered items. In these cases, blank responses were excluded from the analysis for the specific questions they pertained to, but the remaining data from those respondents were included in the overall analysis.

Loneliness was assessed using the three-item version of the UCLA Loneliness Scale [10], with a Japanese version of the scale employed in this study [11]. Participants who answered "Always feel" to at least one of the three items were categorized as "people who feel lonely." These items were "Do you ever feel that you lack social interaction?" (question 22 of the survey); "Do you ever feel left behind?" (question 23 of the survey); and "Do you ever feel isolated from others?" (question 24 of the survey).

The response options for these items were "Never feel that way," "Rarely feel that way," "Sometimes feel that way," or "Always feel that way."

Survey

The survey consisted of 52 questions covering participants' demographic, socioeconomic, and educational background, loneliness, medical institution and pharmacy use, and community activities.

Economic status was assessed by asking participants, "Compared to the general public, how would you rate your household's standard of living (in terms of clothing, food, housing, leisure, etc.)?" Respondents chose from a nine-point scale: (1) upper upper, (2) upper middle, (3) upper lower, (4) middle upper, (5) middle middle, (6) middle lower, (7) lower upper, (8) lower middle, or (9) lower lower.

Medical institution and pharmacy use were determined based on self-reported data obtained from the survey. A full copy of the survey is provided in Appendix 1.

Statistical analyses

Statistical analyses were conducted using IBM SPSS Statistics for Windows, Version 27.0 (Released 2020; IBM Corp., Armonk, NY, USA). To examine associations between loneliness and each study variable, chi-squared tests were used for categorical variables (sex), and Mann-Whitney U tests were used for ordinal variables (e.g., standard of living and perceived health status). A p-value of less than 0.05 was considered statistically significant for all tests.

## Results

Patient characteristics

Of the 3,369 participants, the majority were female respondents (n = 1,882, 55.9%). The respondents represented a wide age range, as shown in Table 1. Most participants were living as part of a couple with a child or children, as a parent (n = 913, 27.1%), alone (n = 829, 24.6%), or as part of a couple without a child (n = 603, 17.9%). In terms of standard of living, the majority of participants were middle middle class (n = 1,140, 33.8%) or middle upper class (n = 908, 27%). Most participants felt that they were either healthy (n = 1,526, 45.3%) or somewhat healthy (n = 1,423, 42.2%). Almost half (n = 1,671, 49.6%) of participants had undergraduate university-level education.

**Table 1:** Characteristics of the study population

		Number of responses	%
Sex of the respondents	Male	1448	43
	Female	1882	55.9
	Other	10	0.3
	Unknown	29	0.9
	Overall	3369	100
Age	15-19 years	164	4.9
	20-24 years	167	5
	25-29 years	369	11
	30-34 years	347	10.3
	35-39 years	383	11.4
	40-44 years	402	11.9
	45-49 years	433	12.9
	50-54 years	408	12.1
	55-59 years	378	11.2
	60-64 years	296	8.8
	Unknown	22	0.7
	Overall	3369	100
Family type	Living alone	829	24.6
	Couple only	603	17.9
	Couple and child (I am the parent)	913	27.1
	A couple and their child (I am the child)	311	9.2
	Single parent and child (I am the parent)	96	2.8
	Single parent and child (I am the child)	163	4.8
	Three-generation households	170	5
	Other	284	8.4
	Overall	3369	100
Standard of living	Upper upper	28	0.8
	Upper middle	104	3.1
	Upper lower	234	6.9
	Middle upper	908	27
	Middle middle	1140	33.8
	Middle lower	488	14.5
	Lower upper	229	6.8
	Lower middle	132	3.9
	Lower lower	76	2.3
	Unknown	30	0.9
	Overall	3369	100
The condition of one's health	Healthy	1526	45.3
	Somewhat healthy	1423	42.2
	Somewhat unhealthy	300	8.9
	Unhealthy	99	2.9
	Unknown	21	0.6
	Total	3369	100
Highest level of education completed	Middle school	34	1
	High school	565	16.8
	Vocational school	457	13.6
	Colleges of technology and junior colleges	356	10.6
	University	1671	49.6
	Graduate school	268	8
	Other	8	0.2
	Unknown	10	0.3
	Total	3369	100

Main results

The responses for each of the three loneliness questions are shown in Table 2. Across all three questions, there were 17 nonresponses (0.5%), resulting in 3,352 valid responses. For the question "Do you ever feel that you lack social interaction?", the most common responses were "Sometimes feel that way" (n = 1,201, 35.8%), "Rarely feel that way" (n = 1,066, 31.8%), and "Never feel that way" (n = 785, 23.4%). Only 300 (9.0%) participants responded "Always feel that way." For the question "Do you ever feel left behind?", the most common responses were "Rarely feel that way" (n = 1,230, 36.7%), "Never feel that way" (n = 1,126, 33.6%), and "Sometimes feel that way" (n = 839, 25.0%). Only 157 (4.7%) participants responded "Always feel that way." For the question "Do you ever feel isolated from others?", the most common responses were "Rarely feel that way" (n = 1,229, 36.7%), "Never feel that way" (n = 1,154, 34.4%), and "Sometimes feel that way" (n = 808, 24.1%). Only 161 (4.8%) participants responded "Always feel that way." Overall, 379 (11.3%) participants answered "Always feel that way" to at least one of the three items.

**Table 2:** Responses to the questions focusing on loneliness

	Number of responses	%
Do you ever feel that you lack social interaction? (Q22)		
- Never feel that way	785	23.4
- Rarely feel that way	1066	31.8
- Sometimes feel that way	1201	35.8
- Always feel that way	300	9
- No response	17	0.5
Do you ever feel left behind? (Q23)		
- Never feel that way	1126	33.6
- Rarely feel that way	1230	36.7
- Sometimes feel that way	839	25
- Always feel that way	157	4.7
- No response	17	0.5
Do you ever feel isolated from others? (Q24)		
- Never feel that way	1154	34.4
- Rarely feel that way	1229	36.7
- Sometimes feel that way	808	24.1
- Always feel that way	161	4.8
- No response	17	0.5

Participants who felt lonely were significantly more likely to be men (chi-squared test value = 14.732, p < 0.001), economically disadvantaged (in terms of living standards compared with the general public; Mann-Whitney U test value = 336954.500, p < 0.001), and perceived their health status as poor (Mann-Whitney U test value = 386908.000, p < 0.001). Furthermore, loneliness was associated with a significantly lower self-reported frequency of medical institution use (chi-squared test value = 8.680, p = 0.003) and pharmacy use (chi-squared test value = 18.400, p < 0.001).

The full set of responses to all survey questions is provided in Appendix 2.

## Discussion

This study evaluated loneliness and potential associated factors through a survey administered to 3,369 individuals in a ward in Tokyo. The study found that 11.3% of survey respondents indicated that they "Always feel that way" in response to one or more of the three survey items related to loneliness. Those who reported feeling lonely were significantly more likely to be men, economically disadvantaged, and perceived their health status as poor. They also reported significantly lower frequencies of medical institution use and, as a particularly novel finding, a lower frequency of pharmacy use.

The finding that men were more likely to experience loneliness warrants further consideration. Previous research has shown that men are more isolated than women [12], suggesting that men tend to meet emotional needs through their spouses/partners, whereas women often rely on their female friends for emotional support [12]. These findings may help explain the higher prevalence of psychological distress among men in our study.

The current study utilized the three-item version of the UCLA Loneliness Scale, which has been validated in previous research and offers practical advantages for large-scale surveys. This shortened version, developed by Hughes et al. [9], has demonstrated good reliability and validity, and its Japanese version has been validated by Igarashi [11]. The choice of the three-item scale was primarily motivated by the need to minimize respondent burden in a comprehensive community survey while maintaining measurement validity.

However, it is important to acknowledge that loneliness is a complex psychological construct that may not be fully captured by a brief measurement tool. While the three-item scale focuses on core aspects of loneliness, namely, social isolation, feeling left out, and lack of companionship, it may not capture the more nuanced dimensions addressed by the full 20-item UCLA Loneliness Scale. This limitation highlights the need for future research comparing the short and long versions of the scale in the Japanese context.

Future research should validate the three-item UCLA Loneliness Scale against the 20-item version, specifically in urban Japanese populations like Tokyo. Such research would help determine whether the abbreviated scale adequately captures the complexity of loneliness in this cultural context and whether it identifies the same at-risk populations as the full scale. This validation would be particularly valuable, given the cultural differences in how loneliness is experienced and expressed across different societies.

These results support some of the findings reported in previous studies and suggest potential barriers to accessing necessary medical and pharmacy services for lonely individuals.

The relationship between loneliness and reduced pharmacy use identified in this study may be explained by several potential mechanisms. First, psychological barriers may play a significant role. Lonely individuals often experience heightened anxiety in social situations and tend to avoid social interactions and interpersonal encounters due to increased social anxiety [13], which could make them reluctant to engage with pharmacy staff. Second, health literacy factors may be involved. The lack of social networks may reduce opportunities for individuals to share health information and receive guidance on medication use [14].

However, our results confirm the association between loneliness and economic disadvantage, aligning with the results of a previous study that also used the three-item version of the UCLA Loneliness Scale. That study found that loneliness was significantly more common among individuals residing in more deprived areas compared to those in more affluent areas [15].

Further research on the relationship between loneliness and medical institution and pharmacy use is needed in the future. In particular, the factors that may influence this relationship (such as sex, economic situation, and other variables shown in the current study) should be analyzed in greater detail. This could help develop more effective support measures for individuals experiencing loneliness. The relationship between loneliness and reduced pharmacy use identified herein is a novel finding and warrants further investigation.

Although "social prescribing" has gained attention as an approach to connecting medical and community resources [16-19], this approach may not be effective, given the current results indicating less access to medical institutions. Rather, interventions aimed at removing barriers to accessing medical services may be necessary. To address barriers to medical institution and pharmacy use for individuals with high levels of loneliness and connect them to appropriate care, a comprehensive approach is required. This approach should include not only medical and nursing services but also social participation and life support. Encouraging small acts of kindness among neighbors may help reduce loneliness, social isolation, and social anxiety while fostering neighborhood relationships, as demonstrated in a recent international randomized controlled trial [20]. At a broader level, the development of policy support for governments worldwide is crucial to addressing loneliness and its effects on health outcomes [21].

We found a significant link between loneliness and decreased medical service use. However, the reasons for this relationship remain unclear. Future research should explore several potential barriers: (1) social stigma, including perceived social judgment associated with loneliness and mental vulnerability, psychological barriers preventing help-seeking behaviors, and fears of being seen as socially isolated or weak; (2) logistical challenges, such as transportation limitations, complex appointment systems, limited access to healthcare information, and difficulties navigating healthcare infrastructure; and (3) psychological barriers, including anxiety about social interactions with healthcare providers, fear of communication and vulnerability, reluctance to discuss personal health concerns, and low self-efficacy in managing healthcare needs.

To comprehensively understand these barriers, we recommend that future studies adopt mixed-method approaches, including qualitative research methods such as in-depth qualitative interviews with individuals experiencing loneliness, phenomenological studies exploring lived experiences, focus group discussions, and participatory research methodologies.

By identifying and understanding these barriers, researchers can develop targeted interventions aimed at improving healthcare access for vulnerable populations.

Study limitations

The following are certain limitations of this study.

Self-Reported Data

The data on medical institution and pharmacy use were based on self-reported information, which may not accurately reflect actual healthcare utilization. A meta-analysis of loneliness and primary healthcare use found that effect sizes were significantly larger when relying on objective data, such as primary care use, rather than self-reported data [22]. Future research should incorporate objective data sources like insurance claims or electronic health records to validate these findings.

Limited Statistical Analysis

Our analyses were limited to bivariate associations and did not account for potential confounding variables such as age, education, or family structure. Multivariate analyses in future studies would provide a more comprehensive understanding of the relationship between loneliness and healthcare use.

Geographic and Sampling Constraints

The study was conducted in a single ward in Tokyo with a low response rate (33.7%), which limits the generalizability of our findings. We could not determine whether nonrespondents systematically differed from participants, potentially introducing nonresponse bias.

Cultural Specificity

The results may be specific to the urban Japanese context. The association between mortality risk and loneliness is culture-related, with one study finding a stronger association in Korea compared to England, the United States, or Mexico [23]. Further research in diverse populations is needed to confirm the generalizability of these findings.

These limitations suggest the need for more comprehensive, multicenter studies that use objective measurement tools and include diverse population samples.

## Conclusions

This study's findings suggest that the feeling of loneliness is associated with male gender, economic disadvantage, lower perceived health status, and lower self-reported frequency of medical institution AND pharmacy use. These results indicate the possibility of barriers preventing lonely individuals from accessing necessary medical and pharmacy services. Further research is needed to explore this intriguing finding in more detail.

## Appendices

Appendix 1: survey questionnaire

Survey on Living Conditions and Awareness in Nakano Ward

1. Questions about yourself

Q1. Please indicate your sex.

a. Male

b. Female

c. Other

Q2. Which of the following age groups do you belong to?

a. 15-19 years

b. 20-24 years

c. 25-29 years

d. 30-34 years

e. 35-39 years

f. 40-44 years

g. 45-49 years

h. 50-54 years

i. 55-59 years

j. 60-64 years

Q3. Which of the following best describes your living situation? (Omitted)

Q4. How many years have you lived in Nakano Ward? (Omitted)

Q5. Including yourself, how many people currently live with you? (Omitted)

Q6. Do you provide daily care for a family member? (Omitted)

Q7. Who is primarily responsible for supporting your household? (Omitted)

Q8. Compared to the general public, how would you rate your household's standard of living (in terms of clothing, food, housing, leisure, etc.)? Please answer based on your personal feelings.

a. Upper upper

b. Upper middle

c. Upper lower

d. Middle upper

e. Middle middle

f. Middle lower

g. Lower upper

h. Lower middle

i. Lower lower

Q9. How do you feel about your current health condition?

a. Healthy

b. Somewhat healthy

c. Somewhat unhealthy

d. Unhealthy

Q10. What is the highest level of education you have completed (including dropout) or are currently enrolled in? (Omitted)

Q11. Which area within Nakano Ward do you live in? (Omitted)

2. Questions about your living situation

Q12. Are you currently employed? (Omitted)

Q13. For those who answered “Not working” in Q12, which of the following best describes your current situation? (Omitted)

Q14. For those who are currently working or have worked before, has your employment status changed since March of this year due to the impact of the coronavirus disease pandemic? (Omitted)

Q15. Please circle all the communication methods that you usually use from the following list. (Omitted)

Q16. Please circle all the Nakano Ward public relations media that you usually see from the following list. (Omitted)

Q17. How often do you use delivery services? (Omitted)

Q18. How do you usually have your meals? (Omitted)

Q19. Do you keep any pets? (Omitted)

Q20. Are you a member of any groups or circles and participate in their activities? (Omitted)

Q21. Have you conversed with the following people in the past month? (Omitted)

Q22. Do you ever feel that you lack social interactions?

a. Never feel that way

b. Rarely feel that way

c. Sometimes feel that way

d. Always feel that way

Q23. Do you ever feel left behind?

a. Never feel that way

b. Rarely feel that way

c. Sometimes feel that way

d. Always feel that way

Q24. Do you ever feel isolated from others?

a. Never feel that way

b. Rarely feel that way

c. Sometimes feel that way

d. Always feel that way

Q25. How often did you feel lonely this past week? (Omitted)

Q26. Who listens to your worries or complaints? (Omitted)

Q27. Other than family, friends, or acquaintances, do you have anyone or any institution to consult with when something happens? (Omitted)

Q28. Do you have any current worries? (Omitted)

Q29. Do you have any concerns about the present or future? Please feel free to write. (Omitted)

Q30. Do you have any hobbies? (Omitted)

Q31. Do you have anything that gives your life meaning? (Omitted)

Q32. How happy do you feel currently? Please answer on a scale of 0-10, where 10 is “very happy” and 0 is “very unhappy.” (Omitted)

3. Questions about consultation services in Nakano Ward

Q33. Are you familiar with the “Community-based Integrated Care System”? (Omitted)

Q34. Are you familiar with the “Health and Welfare Center”? (Omitted)

Q35. Are you familiar with the “Community General Support Center”? (Omitted)

Q36. Are you familiar with the “Community Activity Center”? (Omitted)

Q37. Are you familiar with the “Senior Citizens' Center”? (Omitted)

Q38. Are you familiar with “Commissioned Welfare Volunteers/Child Welfare Volunteers”? (Omitted)

4. Questions about your residence and community

Q39. How satisfied are you with your current residence? (Omitted)

Q40. Please circle all the facilities that you frequently use in your residential area.

a. Convenience stores

b. Supermarkets

c. Individual grocery stores/daily goods stores

d. Post office

e. Bank

f. Cafes/coffee shops/restaurants

g. Izakaya (Japanese-style pub)/bars

h. Medical institutions

i. Dental clinics

j. Pharmacies

k. Barber shops/beauty salons

l. Sports facilities

m. Library

n. Park

o. Karaoke shops

p. Internet cafes

Q41. Do you think there is mutual support and a sense of looking out for each other among residents in your area? (Omitted)

Q42. What kind of connections do you wish to have with people in your residential area? (Omitted)

Q43. Do you want to continue living in Nakano Ward in the future? (Omitted)

5. Questions about community activities and places to belong

Q44. Do you think you have any knowledge, skills, or experiences that could be useful for the community? (Omitted)

Q45. Are you currently using your knowledge, skills, or experiences for the benefit of the community? (Omitted)

Q46. Do you have a “place to belong” in your current residential area other than your home? (Omitted)

Q47. What kind of “places to belong” do you think would be good to have in Nakano Ward? Please select all that apply. (Omitted)

6. Questions about local medical care and consultation places

Q48. Have you decided on a regular clinic (excluding hospitals) near your home for medical examinations and health consultations? (Omitted)

Q49. How do you choose medical institutions when you need to see a doctor? (Omitted)

Q50. Are you aware that there are medical institutions, dental clinics, and pharmacies in Nakano Ward that are open from 9 AM to 5 PM on Sundays and public holidays? (Omitted)

Q51. For each of the following issues, what kind of place do you think would be easy to consult? Please select all that apply. (Omitted)

7. Other

Q52. Currently, Nakano Ward is considering a system where people who have not been interested in community activities before can engage in activities they want to do in the community, thereby energizing the entire community. If you have any ideas about how such a system should be, please feel free to write them down. (Omitted)

Appendix 2: detailed survey results

Survey on Living Conditions and Awareness in Nakano Ward

This survey was distributed to 10,000 randomly selected residents aged 15-64 as of October 1, 2020.

1. Questions about yourself

Q1. Please indicate your sex. Encircle one answer. (Number of respondents: 3,369)

a. Male (43.0%)

b. Female (55.9%)

c. Other (0.3%)

d. No response (0.9%)

Q2. Which age group do you belong to? Encircle one answer. (Number of respondents: 3,369)

a. 15-19 years (4.9%)

b. 20-24 years (5.0%)

c. 25-29 years (11.0%)

d. 30-34 years (10.3%)

e. 35-39 years (11.4%)

f. 40-44 years (11.9%)

g. 45-49 years (12.9%)

h. 50-54 years (12.1%)

i. 55-59 years (11.2%)

j. 60-64 years (8.8%)

k. No response (0.7%)

Q3. Which type of housing do you live in? Encircle one answer. (Number of respondents: 3,369)

a. Owned house (detached) (30.3%)

b. Owned apartment/condominium (18.6%)

c. Rented house (detached) (2.5%)

d. Private rental apartment/condominium (23.2%)

e. Private rental apartment (smaller type) (17.3%)

f. Public housing (1.4%)

g. Company housing/civil servant housing (3.8%)

h. Rented room (0.9%)

i. Other (1.3%)

j. No response (0.7%)

Q4. How long have you lived in Nakano Ward? Encircle one answer. (Number of respondents: 3,369)

a. Less than one year (7.5%)

b. One to less than three years (12.4%)

c. Three to less than five years (9.6%)

d. Five to less than 10 years (17.5%)

e. Ten to less than 20 years (22.4%)

f. Twenty to less than 30 years (14.2%)

g. Thirty years or more (15.7%)

h. No response (0.7%)

Q5. Including yourself, how many people live in your household? Encircle one answer. (Number of respondents: 3,369)

a. Living alone (24.6%)

b. Two people (27.6%)

c. Three people (22.2%)

d. Four people (17.8%)

e. Five or more people (7.2%)

f. No response (0.6%)

Q5-1. For those who answered “two people” to “five or more people,” who lives with you? Encircle all that apply. (Number of respondents: 2,520)

a. Spouse (including common-law marriage) (66.4%)

b. Partner (other than spouse/common-law marriage) (4.1%)

c. Children (including children's spouses) (45.7%)

d. Parent(s) (including in-laws) (27.1%)

e. Sibling(s) (11.7%)

f. Grandparent(s) (including in-laws) (2.5%)

g. Grandchild(ren) (0.4%)

h. Other relatives (0.6%)

i. Friends/acquaintances (1.2%)

j. Other (0.8%)

k. No response (0.3%)

Q6. Do you regularly provide care for family members? Encircle one. (Number of respondents: 3,369)

a. Yes (4.6%)

b. No (94.4%)

c. No response (1.0%)

Q6-1. For those who answered “yes,” who requires care? Encircle all that apply. (Number of respondents: 155)

a. Older person(s) (86.5%)

b. Person(s) with disabilities (including children) (14.2%)

c. Other (5.8%)

d. No response (0.0%)

Q7. Who is primarily responsible for supporting your household's livelihood? If multiple people contribute, please select the person who contributes the most. If you primarily rely on financial support from others, please select the person who provides that support. Encircle one. (Number of respondents: 3,369)

a. Yourself (54.3%)

b. Father (10.5%)

c. Mother (2.9%)

d. Spouse (27.7%)

e. Sibling(s) (0.4%)

f. Child(ren) (0.1%)

g. Other family members or relatives (0.3%)

h. Receiving pension benefits (0.7%)

i. Receiving public assistance (0.9%)

j. Other (1.2%)

k. No response (0.9%)

Q8. How would you rate your standard of living (material living standards including clothing, food, housing, and leisure) compared to the general public? Please answer based on your personal perception. Encircle one. (Number of respondents: 3,369)

a. Upper upper class (0.8%)

b. Upper middle class (3.1%)

c. Upper lower class (6.9%)

d. Middle upper class (27.0%)

e. Middle middle class (33.8%)

f. Middle lower class (14.5%)

g. Lower upper class (6.8%)

h. Lower middle class (3.9%)

i. Lower lower class (2.3%)

j. No response (0.9%)

Q9. How do you feel about your current health condition? Encircle one. (Number of respondents: 3,369)

a. Healthy (45.3%)

b. Somewhat healthy (42.2%)

c. Somewhat unhealthy (8.9%)

d. Unhealthy (2.9%)

e. No response (0.6%)

Q10. What is the highest level of education you have completed (including current enrollment)? Encircle one. (Number of respondents: 3,369)

a. Junior high school (1.0%)

b. High school (16.8%)

c. Vocational school (13.6%)

d. Technical college/Junior college (10.6%)

e. University (49.6%)

f. Graduate school (8.0%)

g. Other (0.2%)

h. No response (0.3%)

Q11. Which area within Nakano Ward do you live in? Encircle one. (Number of respondents: 3,369)

a. Minamidai (5.9%)

b. Yayoicho (6.3%)

c. Honcho (9.9%)

d. Chuo (8.1%)

e. Higashinakano (7.7%)

f. Nakano (9.1%)

g. Kamitakada (5.5%)

h. Arai (6.1%)

i. Numabukuro (4.0%)

j. Matsugaoka (1.0%)

k. Eharacho (2.3%)

l. Egota (3.7%)

m. Maruyama (1.2%)

n. Nogata (5.7%)

o. Yamatocho (5.2%)

p. Wakamiya (3.6%)

q. Shirasagi (3.7%)

r. Saginomiya (5.2%)

s. Kamisaginomiya (5.1%)

t. No response (0.6%)

2. Questions about your living conditions

Q12. Are you currently employed? Encircle one. (Number of respondents: 3,369)

a. Employed (80.3%)

b. Not employed (19.4%) → Go to Q13

c. No response (0.3%)

Q12-1. For those who answered “employed,” which of the following best describes your employment status? Encircle one. If multiple applies, choose the main one. (Number of respondents: 2,706)

a. Regular employee/staff member (59.7%)

b. Contract worker/temporary staff/part-time worker/casual worker (25.0%)

c. Company executive/officer (3.6%)

d. Self-employed with employees (2.2%)

e. Self-employed without employees (6.9%)

f. Family worker in the family business (1.0%)

g. Other (1.4%)

h. No response (0.2%)

Q12-2. For those who answered “employed,” do you currently have any side jobs? Encircle one. (Number of respondents: 2,706)

a. Yes (6.8%)

b. No (92.7%)

c. No response (0.5%)

Q12-3. For those who answered “employed,” how has your work style changed since March of this year due to the coronavirus disease 2019 (COVID-19) pandemic? Encircle all that apply. (Number of respondents: 2,706)

a. Completely shifted to working from home (12.3%)

b. Partially shifted to working from home (32.6%)

c. Meetings and discussions became primarily online (21.8%)

d. Business/visit activities became primarily non-face-to-face (phone, email, etc.) (7.4%)

e. Some online meetings were introduced (11.9%)

f. No change from before (42.3%)

g. No response (1.4%)

Q12-4. For those who answered “employed,” has your income changed due to the COVID-19 pandemic? Encircle one. (Number of respondents: 2,706)

a. Significantly decreased (12.3%)

b. Slightly decreased (27.2%)

c. No change (57.1%)

d. Slightly increased (2.4%)

e. Significantly increased (0.3%)

f. No response (0.6%)

Q13. For those who answered “not employed” in Q12, which of the following describes your current situation? Encircle one. (Number of respondents: 654)

a. Student (30.9%)

b. Full-time homemaker (39.8%)

c. Helping with family housework (2.1%)

d. Unemployed (not looking for work) (7.3%)

e. Unemployed (currently job hunting) (12.8%)

f. Other (7.0%)

g. No response (0.0%)

Q14. For those who are currently working or have worked before, has your employment status changed since March of this year due to the COVID-19 pandemic? Encircle one. (Number of respondents: 3,369)

a. Lost my job (2.6%)

b. Employment status changed (e.g., from full-time to part-time) (0.9%)

c. Same employment status but experienced changes such as reduced working days (13.6%)

d. Other changes (please specify:) (6.9%)

e. No change from before (65.8%)

f. No response (10.2%)

Q15. Which of the following communication methods do you regularly use? Encircle all that apply. (Number of respondents: 3,369)

a. Landline telephone (29.7%)

b. Fax (12.3%)

c. Mobile phone calls (including calls via apps such as LINE) (88.9%)

d. Mobile phone text messages (72.6%)

e. PC email (51.3%)

f. Chat (including apps such as LINE) or messenger services (61.6%)

g. Website browsing/posting (including bulletin boards and blogs) (25.1%)

h. Social Networking Services (Facebook, mixi, LinkedIn, etc.) browsing/posting (28.9%)

i. Twitter (33.4%)

j. Instagram (33.5%)

k. Online games (15.6%)

l. Other (0.7%)

m. No response (0.9%)

Q16. Which of the following Nakano Ward public relations media do you usually see? Encircle all that apply. (Number of respondents: 3,369)

a. “Nakano Ward News” (municipal newsletter) (73.6%)

b. Ward bulletin boards (10.5%)

c. Ward website (20.1%)

d. Ward Facebook page (0.8%)

e. Ward Twitter account (3.0%)

f. Ward YouTube official channel (0.2%)

g. Other (0.7%)

h. Have not seen any (19.6%)

i. No response (1.2%)

Q17. How often do you use delivery services? Note: This includes all deliveries such as items purchased online and food delivery services. Encircle one. (Number of respondents: 3,369)

a. Daily (0.6%)

b. Five to six times per week (1.4%)

c. Three to four times per week (6.0%)

d. One to two times per week (21.4%)

e. Two to three times per month (28.6%)

f. Once a month (14.8%)

g. Once every few months (12.5%)

h. Once or twice a year (4.7%)

i. Do not use (9.3%)

j. No response (0.7%)

Q18. How do you usually handle your meals? Fill in the number for each. (Number of respondents: 3,369)

(1) Breakfast

a. Cook at home (or eat meals prepared by family members) (68.3%)

b. Buy prepared food or boxed meals (16.1%)

c. Eat out (4.2%)

d. Other (3.5%)

e. Do not eat breakfast (18.0%)

f. No response (2.9%)

(2) Lunch

a. Cook at home (or eat meals prepared by family members) (66.0%)

b. Buy prepared food or boxed meals (48.2%)

c. Eat out (36.2%)

d. Other (6.3%)

e. Do not eat lunch (3.4%)

f. No response (2.5%)

(3) Dinner

a. Cook at home (or eat meals prepared by family members) (86.9%)

b. Buy prepared food or boxed meals (38.6%)

c. Eat out (33.8%)

d. Other (1.8%)

e. Do not eat dinner (0.6%)

f. No response (2.8%)

Q19. Do you have any pets? Encircle one. (Number of respondents: 3,369)

a. Yes (19.1%)

b. No (80.3%)

c. No response (0.6%)

Q19-1. For those who answered “yes,” what kind of pets do you have? Encircle all that apply. (Number of respondents: 644)

a. Dog(s) (42.9%)

b. Cat(s) (33.2%)

c. Other (30.7%)

d. No response (0.2%)

Q20. Are you a member of any groups or circles? Encircle all that apply. (Number of respondents: 3,369)

a. Volunteer groups (3.0%)

b. Hobby groups (11.1%)

c. Study/learning groups (2.9%)

d. Other groups (2.2%)

e. Not a member of any groups (81.7%)

f. No response (1.3%)

Q21. Have you had conversations with the following people in the past month?  Note: “Conversation” here refers to face-to-face or telephone conversations and does not include text-based communication such as LINE messages or emails. Encircle one for each. (Number of respondents: 3,369)

(1) Family members/household members:

a. Did not converse at all (8.2%)

b. Rarely conversed (5.1%)

c. Sometimes conversed (16.2%)

d. Frequently conversed (69.0%)

e. No response (1.5%)

(2) Neighbors

a. Did not converse at all (48.1%)

b. Rarely conversed (26.7%)

c. Sometimes conversed (22.2%)

d. Frequently conversed (2.0%)

e. No response (1.0%)

(3) Friends/acquaintances

a. Did not converse at all (10.9%)

b. Rarely conversed (16.3%)

c. Sometimes conversed (43.4%)

d. Frequently conversed (28.9%)

e. No response (0.5%)

Q22. Do you ever feel that you lack social interaction? Encircle one. (Number of respondents: 3,369)

a. Never feel that way (23.3%)

b. Rarely feel that way (31.7%)

c. Sometimes feel that way (35.7%)

d. Always feel that way (8.9%)

e. No response (0.4%)

Q23. Do you ever feel left behind? Encircle one. (Number of respondents: 3,369)

a. Never feel that way (33.4%)

b. Rarely feel that way (36.5%)

c. Sometimes feel that way (24.9%)

d. Always feel that way (4.7%)

e. No response (0.5%)

Q24. Do you ever feel isolated from others? Encircle one. (Number of respondents: 3,369)

a. Never feel that way (34.3%)

b. Rarely feel that way (36.5%)

c. Sometimes feel that way (24.0%)

d. Always feel that way (4.8%)

e. No response (0.4%)

Q25. How often did you feel lonely during the past week? Encircle one. (Number of respondents: 3,369)

a. Not at all (68.8%)

b. One to two times during the week (23.0%)

c. Three to four times during the week (4.0%)

d. Almost every day (3.6%)

e. No response (0.6%)

Q26. Who listens to your concerns and complaints? Encircle all that apply. (Number of respondents: 3,369)

a. Spouse/partner (51.6%)

b. Children (13.6%)

c. Parents/grandparents (32.4%)

d. Siblings (18.4%)

e. Other relatives (3.8%)

f. Friends/acquaintances (55.7%)

g. Colleagues at work (31.6%)

h. Members of groups you belong to (2.4%)

i. Online friends/bulletin board users (3.0%)

j. Fortune tellers/spiritual counselors (0.5%)

k. Other (1.4%)

l. No one (7.3%)

m. No response (0.5%)

Q27. Other than family members and friends, do you have anyone or any institutions you can consult with when you have problems? Encircle all that apply. (Number of respondents: 3,369)

a. Neighborhood associations/community associations (1.8%)

b. Community welfare commissioners/child welfare volunteers (0.7%)

c. Social welfare council (0.8%)

d. Government offices and other public institutions (8.9%)

e. Psychiatrists, clinical psychologists, or other mental health professionals (7.4%)

f. Medical doctors (non-psychiatric) or other professionals such as lawyers (4.8%)

g. Other (3.8%)

h. No one (75.5%)

i. No response (1.7%)

Q27-1. For those who answered “no one,” why do you not consult with anyone? Encircle all that apply. (Number of respondents: 2,542)

a. Do not want others to know about my personal matters (8.7%)

b. Do not think consultation would solve the problem (20.6%)

c. Anxious about what I might be asked (4.1%)

d. Feel I cannot explain well/will not be understood (9.2%)

e. Do not want others to know that I sought consultation (3.6%)

f. Think it would cost money (10.2%)

g. No consultation services nearby (4.6%)

h. Do not know who to consult with (29.0%)

i. Do not have the energy to seek consultation (4.8%)

j. Other (11.3%)

k. No particular reason (40.3%)

l. No response (0.4%)

Q28. Do you currently have any concerns? Encircle all that apply. (Number of respondents: 3,369)

a. Work-related concerns (29.0%)

b. Work-life balance (10.3%)

c. Financial issues (29.4%)

d. Child-raising/nursing care (11.1%)

e. Health of self or family members (23.0%)

f. Family relationships (9.8%)

g. Relationships outside the family (6.9%)

h. Housing issues (14.8%)

i. Problems with neighbors (2.3%)

j. Academic/educational issues (7.1%)

k. Concerns about appearance/looks (10.2%)

l. Concerns about personality (10.0%)

m. Purpose in life/self-worth issues (16.3%)

n. Other (5.2%)

o. No particular concerns (25.6%)

p. No response (1.4%)

Q29. Do you have any anxieties about your present or future situation? Please describe freely.

Q30. Do you have any hobbies? Encircle one. (Number of respondents: 3,369)

a. Yes (74.7%)

b. No (24.1%)

c. No response (1.2%) 

Q31. Do you have ikigai (purpose in life/something that makes life worth living)? Encircle one. (Number of respondents: 3,369)

a. Yes (67.1%)

b. No (31.0%)

c. No response (1.9%)

Q32. How happy do you currently feel? Please rate your happiness on a scale of 0-10, where 10 means “very happy” and 0 means “very unhappy.” Encircle one. (Number of respondents: 3,369)

a. 0 pt (0.4%)

b. 1 pt (1.0%)

c. 2 pts (1.8%)

d. 3 pts (4.4%)

e. 4 pts (4.6%)

f. 5 pts (14.5%)

g. 6 pts (10.4%)

h. 7 pts (19.3%)

i. 8 pts (22.5%)

j. 9 pts (9.3%)

k. 10 pts (10.4%)

l. No response (1.5%)

3. Questions about consultation services in Nakano Ward

Q33. Are you familiar with the “Community-based Integrated Care System”? Encircle one. (Number of respondents: 3,369)

Community-based Integrated Care System: This is a system that enables all ward residents to continue living their lives as they wish in their familiar community until the end of life, by providing integrated housing, medical care, nursing care, health promotion, and life support services. Currently, Nakano Ward is working with related organizations to establish this system.

a. Well aware of it (3.2%)

b. Somewhat aware of it (9.9%)

c. Have heard of it but do not know much about it (21.3%)

d. Never heard of it (64.8%)

e. No response (0.8%)

Q34. Are you familiar with the “Sukoyaka Fukushi Center” (Community Health and Welfare Center)? Encircle one. (Number of respondents: 3,369)

Sukoyaka Fukushi Center: The Sukoyaka Fukushi Center is a community-based facility that provides comprehensive support to ensure all residents, including children, older people, and people with disabilities, can live with peace of mind. There are four locations in the ward, where professional staff including public health nurses provide consultations on physical and mental health, child-raising, and child development.

a. Well aware of it (18.0%)

→ Usage experience. Encircle one. (Number of respondents: 605)

(i) Zero times (8.4%)

(ii) One to two times (37.4%)

(iii) Three to five times (33.9%)

(iv) Six to ten times (9.8%)

(vi) 11 or more times (7.3%)

(vii) No response (3.3%)

b. Have heard of it but do not know much about it (28.1%)

c. Never heard of it (52.4%)

d. No response (1.6%)

Q35. Are you familiar with the “Community General Support Center”? Encircle one. (Number of respondents: 3,369)

Community General Support Center: The Community General Support Center is a comprehensive consultation service for geriatric care, health, and welfare. There are eight locations in the ward where health and welfare professionals provide consultations not only for long-term care insurance applications but also for daily life issues of older people and concerns of family caregivers.

a. Well aware of it (10.5%)

→ Usage experience. Encircle one. (Number of respondents: 355)

(i) Zero times (27.9%)

(ii) One to two times (49.9%)

(iii) Three to five times (10.1%)

(iv) Six to ten times (2.0%)

(v) Eleven or more times (3.7%)

(vi) No response (6.5%)

b. Have heard of it but do not know much about it (26.8%)

c. Never heard of it (61.4%)

d. No response (1.3%)

Q36. Are you familiar with the “Community Activity Center”? Encircle one. (Number of respondents: 3,369)

Community Activity Center: There are 15 Community Activity Centers in the ward that serve as activity hubs for residents' voluntary and independent initiatives. In addition to providing meeting rooms for rent, they also serve as “general consultation counters” where residents can discuss their concerns and problems.

a. Well aware of it (21.2%)

→ Usage experience. Encircle one. (Number of respondents: 714)

(i) Zero times (12.2%)

(ii) One to two times (29.7%)

(iii) Three to five times (32.5%)

(iv) Six to ten times (4.1%)

(v) Eleven or more times (18.1%)

(vi) No response (3.5%)

b. Have heard of it but do not know much about it (34.3%)

c. Never heard of it (43.6%)

d. No response (0.9%)

Q37. Are you familiar with the “Senior Citizens' Center”? Encircle one. (Number of respondents: 3,369)

Senior Citizens' Center: There are 16 Senior Citizens' Centers in the ward, available for use by residents aged 60 years and older. They provide meeting rooms and implement health promotion and care prevention programs to support community interaction and independent activities.

a. Well aware of it (6.9%)

→ Usage experience (Circle one): <Number of respondents: 234>

(i) Zero times (44.0%)

(ii) One to two times (33.3%)

(iii) Three to five times (8.5%)

(iv) Six to ten times (2.1%)

(v) Eleven or more times (6.0%)

(vi) No response (6.0%)

b. Have heard of it but do not know much about it (27.2%)

c. Never heard of it (65.3%)

d. No response (0.5%)

Q38. Are you familiar with “Commissioned Welfare Volunteers/Child Welfare Volunteers”? Encircle one. (Number of respondents: 3,369)

Commissioned Welfare Volunteers/Child Welfare Volunteers: These volunteers are appointed by the Minister of Health, Labour and Welfare to provide consultation on welfare-related issues including daily life difficulties and child-raising concerns of local residents. They are bound by legal confidentiality obligations, ensuring that consultations remain private.

a. Well aware of it (14.3%)

b. Have heard of it but do not know much about it (45.1%)

c. Never heard of it (40.2%)

d. No response (0.4%)

4. Questions about your residence and community

Q39. How satisfied are you with your current residence? Encircle one. (Number of respondents: 3,369)

a. Very satisfied (19.8%)

b. Somewhat satisfied (63.1%)

c. Somewhat dissatisfied (13.4%)

d. Very dissatisfied (3.1%)

e. No response (0.7%)

Q40. Which of the following do you frequently use in your local area? Encircle all that apply. (Number of respondents: 3,369)

a. Convenience stores (82.6%)

b. Supermarkets (91.4%)

c. Individual food/daily necessities shops (20.6%)

d. Post office (47.0%)

e. Bank (46.4%)

f. Cafes/coffee shops/restaurants (27.7%)

g. Izakaya (Japanese-style pub)/bars (16.4%)

h. Medical facilities (46.6%)

i. Dental clinics (31.9%)

j. Pharmacies (57.4%)

k. Barber shops/beauty salons (32.8%)

l. Sports facilities (9.3%)

m. Libraries (19.8%)

n. Parks (25.6%)

o. Karaoke venues (4.3%)

p. Internet cafes (0.8%)

q. Other (1.5%)

r. No response (0.4%)

Q41. Do you think there is mutual support and monitoring among residents in your area? Encircle one. (Number of respondents: 3,369)

a. Very much so (1.6%)

b. Somewhat (21.2%)

c. Not very much (19.9%)

d. Not at all (14.1%)

e. Do not know (42.7%)

f. No response (0.5%)

Q42. What kind of relationship would you like to have with people in your neighborhood? Encircle all that apply. (Number of respondents: 3,369)

a. Close enough to discuss personal concerns (5.8%)

b. Close enough to ask for child care or pet sitting (7.5%)

c. Close enough to ask for simple household help such as taking out garbage (5.0%)

d. Close enough to share prepared meals or groceries (6.8%)

e. Close enough for casual conversation (31.2%)

f. Knowing names and family composition (17.2%)

g. Close enough to exchange greetings (75.0%)

h. Do not want any relationship (12.6%)

i. Other (2.2%)

j. No response (0.9%)

Q43. Do you want to continue living in Nakano Ward? Encircle one. (Number of respondents: 3,369)

a. Want to continue living here (61.4%)

b. Want to move but have to stay (7.3%)

c. Want to move out of the ward but have not decided when (7.2%)

d. Plan to move out of the ward soon (3.2%)

e. Do not know (20.5%)

f. No response (0.4%)

Q43-1. For those who answered “want to move but have to stay,” “want to move out,” or “plan to move,” what are your reasons for wanting to move out of Nakano Ward? Encircle all that apply. (Number of respondents: 597)

a. High rent/mortgage payments (30.0%)

b. Small housing (34.8%)

c. Inconvenient for commuting/schooling (9.5%)

d. Poor living environment for shopping, hospitals, etc. (9.4%)

e. Poor environment for child-raising/education (11.2%)

f. Lack of nature (21.1%)

g. Poor public safety (11.1%)

h. Job transfer or other work-related reasons (10.2%)

i. Family reasons such as caring for parents (6.7%)

j. Higher risk of infectious diseases such as COVID-19 in urban areas (5.2%)

k. Poor neighborhood relationships (6.0%)

l. Other (27.6%)

m. No response (1.3%)

5. Questions about community activities and places to belong

Q44. Do you have any knowledge, skills, or experience that you think could benefit the local community? Encircle all that apply. (Number of respondents: 3,369)

a. Language skills (13.6%)

b. IT/computer skills (21.3%)

c. Professional knowledge/skills in law, accounting, etc. (5.4%)

d. Artistic skills in music, painting, etc. (12.6%)

e. Practical skills in cooking, sewing, crafts, etc. (9.3%)

f. Knowledge/skills in childcare and child-raising (8.0%)

g. Skills in teaching/tutoring children (12.6%)

h. Knowledge/skills in gardening or agriculture (1.2%)

i. Knowledge/skills in sports and recreation (9.9%)

j. Knowledge/skills in healthcare, nursing care, or public health (8.5%)

k. Physical labor skills (for carrying items, setting up venues, etc.) (8.5%)

l. Other (18.3%)

m. No response (14.9%)

Q45. Are you currently using your knowledge, skills, or experience to benefit the local community? Encircle one. (Number of respondents: 3,369)

a. Yes (4.6%)

b. No (92.6%) → Go to Q45-2

c. No response (2.8%)

Q45-1. For those who answered “yes,” please specify.

(1) What kind of knowledge/skills you have

(2) How you are using them

Example:

(1) Type of knowledge/skills: English conversation

(2) How you use them: Teaching children as a volunteer

Q45-2. For those who answered “no,” under what conditions do you think you could use your knowledge or skills to benefit the local community? Encircle all that apply. (Number of respondents: 3,119)

a. If reasonable compensation were provided (32.5%)

b. If there were a suitable venue for activities (28.2%)

c. If there were others to work with (17.6%)

d. If support from government (training, matching with activity groups, etc.) were available (15.6%)

e. If I had more free time (56.1%)

f. Not interested in using skills for community benefit under any conditions (5.9%)

g. Other (12.3%)

h. No response (2.1%)

Q46. Do you have a “place to belong” in your local community other than your home? Note: There is no strict definition of “place to belong” here. Please answer based on your own understanding of what constitutes such a place. Encircle one. (Number of respondents: 3,369)

a. Yes (38.7%)

b. No (59.9%)

c. No response (1.4%)

Q46-1. For those who answered “yes,” please describe specifically what kind of place serves as your “place to belong.”

Q47. What kind of “places to belong” would you like to have in Nakano Ward? Encircle all that apply. (Number of respondents: 3,369)

a. Located close to home (45.6%)

b. Free to use (49.7%)

c. Open to anyone (no registration required) (30.6%)

d. Limited to specific users (9.9%)

e. Can stay as long as desired (24.5%)

f. Can do whatever you like (27.8%)

g. Has simple activities available (16.7%)

h. Has professionals available for consultation (17.7%)

i. Opportunities to help others (18.3%)

j. Can obtain information (32.4%)

k. No need to do anything specific (19.3%)

l. Can chat with others (18.7%)

m. Stylish interior/atmosphere (23.8%)

n. Other (6.9%)

o. No response (4.7%)

6. Questions about local medical care and consultation places

Q48. Do you have a regular clinic (excluding hospitals) near your home for medical consultations and health check-ups? Encircle one. (Number of respondents: 3,369)

a. Yes (31.1%)

b. Generally yes (30.9%)

c. No (37.5%)

d. No response (0.5%)

Q49. How do you choose medical facilities when seeking treatment? Encircle all that apply. (Number of respondents: 3,369)

a. Local medical facilities (71.6%)

b. Recommendations/reviews from family or acquaintances (33.7%)

c. Online reviews (38.8%)

d. Home care consultation counter at ward office (0.1%)

e. Nakano Medical Association's family doctor referral service (0.9%)

f. Other (5.1%)

g. No response (0.9%)

Q50. Are you aware that there are medical facilities, dental clinics, and pharmacies in Nakano Ward that are open from 9:00 AM to 5:00 PM on Sundays and holidays? Note: Information about “Holiday Duty Medical Facilities and Pharmacies” is published on the Nakano Ward website and in the “Nakano Ward News” issued on the 5th and 20th of each month. Encircle one. (Number of respondents: 3,369)

a. Yes (60.4%)

b. No (39.0%)

c. No response (0.5%)

Q51. What kind of places would you feel comfortable consulting about each of the following issues? Please select all that apply for each category. (Number of respondents: 3,369)

(1) Financial issues

a. Public institutions such as ward offices (34.6%)

b. Private organizations such as NPOs (9.9%)

c. Has professional staff (44.7%)

d. Free consultation available (53.7%)

e. Can consult anonymously (36.5%)

f. Consultation sessions at events (3.9%)

g. Staff who listen sympathetically (29.2%)

h. Can meet others with similar concerns (8.3%)

i. Close to home (27.6%)

j. Government staff who can visit your home (4.1%)

k. Private professionals who can visit your home (3.9%)

l. Can consult by phone or online (29.4%)

m. Can consult via email or internet (33.8%)

m. Other (1.7%)

o. Do not want to consult anywhere (8.7%)

p. No response (1.4%)

(2) Family and relationship issues

a. Public institutions such as ward offices (18.0%)

b. Private organizations such as NPOs (11.2%)

c. Has professional staff (39.1%)

d. Free consultation available (43.3%)

e. Can consult anonymously (39.3%)

f. Consultation sessions at events (3.0%)

g. Staff who listen sympathetically (34.3%)

h. Can meet others with similar concerns (14.5%)

i. Close to home (19.8%)

j. Government staff who can visit your home (3.4%)

k. Private professionals who can visit your home (3.5%)

l. Can consult by phone or online (27.5%)

m. Can consult via email or internet (32.7%)

n. Other (1.9%)

o. Do not want to consult anywhere (13.4%)

p. No response (2.7%)

(3) Employment, workplace, and career issues

a. Public institutions such as ward offices (32.2%)

b. Private organizations such as NPOs (14.0%)

c. Has professional staff (49.2%)

d. Free consultation available (44.4%)

e. Can consult anonymously (25.0%)

f. Consultation sessions at events (7.9%)

g. Staff who listen sympathetically (26.5%)

h. Can meet others with similar concerns (10.1%)

i. Close to home (19.8%)

j. Government staff who can visit your home (2.3%)

k. Private professionals who can visit your home (2.8%)

l. Can consult by phone or online (27.0%)

m. Can consult via email or internet (33.5%)

n. Other (1.8%)

o. Do not want to consult anywhere (10.1%)

p. No response (3.1%)

(4) Various other life issues

a. Public institutions such as ward offices (21.4%)

b. Private organizations such as NPOs (12.7%)

c. Has professional staff (40.5%)

d. Free consultation available (43.2%)

e. Can consult anonymously (34.9%)

f. Consultation sessions at events (4.2%)

g. Staff who listen sympathetically (32.6%)

h. Can meet others with similar concerns (15.6%)

i. Close to home (19.4%)

j. Government staff who can visit your home (3.1%)

k. Private professionals who can visit your home (3.2%)

l. Can consult by phone or online (27.6%)

m. Can consult via email or internet (31.8%)

n. Other (1.7%)

o. Do not want to consult anywhere (13.7%)

p. No response (3.1%)

7. Other

Q52. Currently, Nakano Ward is considering ways to revitalize the community by encouraging people who have not previously been interested in community activities to engage in activities they would like to do. If you have any ideas about how such a system should work, please feel free to describe them.
